# Supplementary material for: Structural and Neuronal Integrity Measures of Fatigue Severity in Multiple Sclerosis
Source: Brain Sci. 2017 Aug 12;7(8):102. doi: 10.3390/brainsci7080102 (PMC5575622; doi:10.3390/brainsci7080102)
Supplement: Supplementary file 1 [file brainsci-07-00102-s001.pdf]

Supplementary Table S1: Bonferroni Multiple Comparisons. LF—Low Fatigue, HF—High Fatigue, FA—Fractional Anisotropy, RD—Radial Diffusivity.

			p-value	
Bonferroni-Multiple Comparisons			Corrected	Uncorrected
Thalamic volume	LF	HF	*0.007*	*0.001*
Putamen volume	LF	HF	0.46	0.09
Pallidal volume	LF	HF	0.172	*0.013*
Hippocampal volume	LF	HF	1	0.25
Amygdala volume	LF	HF	0.067	0.29
Accumbens volume	LF	HF	0.212	0.06
SCP volume	LF	HF	0.173	*0.002*
FA-Left frontal cortex	LF	HF	1	0.46
FA-Left occipital cortex	LF	HF	1	0.56
FA-Left parietal lobe	LF	HF	1	0.4
FA-Left temporal lobe	LF	HF	0.561	0.2
FA-Right frontal cortex	LF	HF	1	0.7
FA-Right occipital cortex	LF	HF	0.82	0.23
FA-Right parietal cortex	LF	HF	1	0.9
FA-Right temporal cortex	LF	HF	*0.005*	*0.004*
RD-Left frontal cortex	LF	HF	1	0.85
RD-Left occipital cortex	LF	RF	0.637	0.28
RD-Left parietal cortex	LF	HF	1	0.48
RD-Left temporal cortex	LF	HF	0.941	0.43
RD-Right Frontal cortex	LF	HF	0.722	0.21
RD-Right Occipital cortex	LF	HF	0. 297	0.14
RD-Right Parietal cortex	LF	HF	0.411	0.17
RD-Right Temporal cortex	LF	HF	*0.026*	*0.016*
